# Anticancer Activity a of Caspian Cobra (*Naja naja oxiana*) snake Venom in Human Cancer Cell Lines Via Induction of Apoptosis

**Published:** 2016

**Authors:** Karim Ebrahim, Hossein Vatanpour, Abbas Zare, Farshad H. Shirazi, Mryam Nakhjavani

**Affiliations:** a *Department of Toxicology, School of Pharmacy, Shahid Beheshti University of Medical Sciences, Tehran, Iran. *; b *Razi Vaccine and Serum Research Institute, Karaj-Iran. *; c *Pharmaceutical Sciences Research Center, Shahid Beheshti University of Medical Sciences, Tehran, Iran.*

**Keywords:** Snake, Venom, Aapoptosis, Cytotoxicity, Cancer

## Abstract

Cancer is the leading cause of death worldwide. Current anticancer drugs involve various toxic side effects; efforts are ongoing to develop new anticancer agents especially from the screening of natural compounds. Present study investigated cytotoxic effects and mode of cell death induced by the Caspian cobra venom in some human cancer cell lines.

Cytotoxic effects of snake venom toxins (SVT) were investigated via monitoring of morphological changes, MTT, trypan blue exclusion and LDH release assays. Mechanism of cell death was determined by AO/EtBr double staining, caspase-3 activity assay, flow cytometric analysis of apoptosis and mitochondrial membrane potential measurement.

In morphological analysis, apoptotic alterations related to apoptosis such as cytoplasmic blebbing, chromatin condensation and irregularity in shape were seen. IC_50_ of SVT in HepG2, MCF7and DU145 cell lines were 26.59, 28.85 and 21.17µg/mL, respectively and significantly different from the MDCK normal cell line (IC_50_=47.1 µg/mL). AO/EtBr double staining showed the best apoptotic/necrotic ratio at 15 µg/mL after 48 h. LDH release showed no significant differences between 10 µg/mL SVT and cisplatin. Flowcytometric analysis confirms mitochondrial membrane potential loss and more than 95% apoptotic cell death at 15 µg/mL. Caspase-3 was significantly activated at doses higher than 2.5 μg/mL with a maximal activity at 10 μg/mL.

Results from this study demonstrate that SVT induces mitochondrial and caspase-3 dependent apoptosis in cancer cell lines with minimum effects on studied normal cell. This potential might candidate this venom as a suitable choice for cancer treatment

## Introduction

Cancer is a group of neoplastic diseases that occur in human and all other species of animal. Tumor cells are characterized by uncontrolled growth, invasion and metastasis to the surrounding tissues and distant organs. Mortality from cancer is often a consequence of metastasis (1). Cancer is a the leading cause of death worldwide and about 12.7 million cancer cases and 7.6 million cancer deaths are estimated to occur annually (2).

An ideal anticancer agent must kill tumor cells without harming normal cells. Unfortunately, current anticancer drugs involve various toxic side effects that decrease the therapeutic indexes of these agents (1). Efforts are thus ongoing to develop effective and safe anticancer drugs through the screening of natural compounds as well as the syntheses of new chemical compounds (3). 

Snake venom toxins (SVT) is a complex mixture of proteins and peptides and its use as a drug has been known for many decades. However, the curative properties of the snake venoms are always covered by their high toxicities. Study of snake venom as an antitumor agent dates back to the beginning of the past century. Calmette and colleagues were the first group to report the antitumor effects of cobra venom on the adenocarcinoma cells (4). In later years, several studies showed that snake venoms can inhibit the growth and proliferation of various types of cancer cells (5-6). 

Snake venoms exert their anticancer effects via different mechanisms such as: blocking some specific ion channels (5), inhibition of angiogenesis (7) and activating intracellular pathways inducing apoptosis (8-9). Since the cytotoxic effects of venoms on normal and malignant cells have mostly led to similar results in many investigations, researches are continuing on various types of snake venom and their fractions hoping for identification of a crude venom or a venom fraction suitable for cancer treatment with minimum toxic effects on normal cells (10).

The Caspian cobra (*Naja naja oxiana*), also called the Central Asian cobra or Oxus cobra, is a highly venomous species of cobra in the family Elapidae found in Central Asia. Its venom is rich of neurotoxins, cytotoxins and cardiotoxins (11). Studies of Strizhkov and colleagues in 1994 showed that neurotoxin II from the venom of *Naja naja oxiana* can induce apoptosis in the mouse fibroblast (L929) and the human erythroleukemic (KS62) cell lines (12). This result was obtained by only a DNA fragmentation assay and no information was presented on the mode of death induced by this venom on these cell lines. Another study on Caspian cobra venom cytotoxins revealed that Cytotoxins I and II easily penetrate into the living cancer cells and accumulate markedly in the lysosomes, suggesting the lysosomal damage to be the cause of cell death induced by these toxins (13).

The aim of the present study is to further investigation on the cytotoxicity and mode of cell death caused by the venom of Caspian cobra against three human cancer cell lines (human breast cancer (MCF-7), Human hepatocellular carcinoma (HepG2) and human prostate carcinoma (DU145) cell lines) using various techniques.

## Exprimental

*Snake venom collection*

Extraction of venom from the snake’s venomous gland was performed in the Department of Venomous Animals and Antivenom Production, Razi Vaccine and Serum Research Institute, Karaj-Iran by allowing the snake to bite into parafilm stretched over a glass cup. Extracted venom was centrifuged for 10 min at 500*g*. The venom was frozen in a −80 °C deep freezer for 4 h and then lyophilized. Working concentrations of venom were prepared freshly by diluting the lyophilized venom in culture media on the days of experiments (14). This study was approved by the ethics committee of Shahid Beheshti University of medical sciences and Razi Vaccine and Serum Research Institute.

*Cell lines and cell culture*

human breast cancer (MCF-7), Human hepatocellular carcinoma (HepG2) and human prostate carcinoma (DU145) cell lines as well as a normal cell line (normal dog kidney cell line; MDCK) were obtained from National Cell bank of Iran (NCBI) affiliated to Pasteur Institute of Iran. Cells were cultured in RPMI1640 (Gibco, Paisley, UK) supplemented with 10% fetal bovine serum (Gibco, Paisley, UK) and penicillin/streptomycin (100 unit/mL) under the humidified atmosphere of 5% CO_2_ and 95% air at 37 °C (15).

*Cell Morphology Analysis*

The cells were treated with different concentrations of SVT for 24, 48 and 72 h and morphological alterations were investigated using a normal inverted light microscopy (Euromex, Holland) equipped with a digital camera (Moticam Pro 2828, China).

*Cell proliferation assays*

The half maximal inhibitory concentration (IC_50_) values of venom on different cell lines were evaluated using MTT (3-(4, 5-dimethylthiazol-2-yl)-2, 5-diphenyltetrazolium bromide) assay. The cells were grown (10^4^ cells/well) in 96-well plates for 24 h at 75% confluency. Cells were then treated with different concentrations of SVT for 24, 48 and 72 h. At the end of treatment, cells were exposed to MTT (5 mg/mL) and incubated at 37 °C for 4 h. After the removal of supernatant, 100 µL of DMSO was added to each well and the plates were left incubated for 10 min to solubilize the formazan crystals. Absorbance for each well of plates was measured using a microplate reader (BioTek-EL808, United States) at a test wavelength of 595 nm and a reference wavelength of 690 nm. The optical density (O.D.) was calculated as absorbance at the reference wavelength minus that of the test wavelength (16). 

*Trypan blue exclusion assay*

To study the cytotoxic effects of SVT and determine viable cell numbers, the cells were seeded in 12-well plates (3×10^4^ cells/well). The cells were treated with different concentrations of venom for 24, 48 and 72 h. At the end of treating time, the cells were harvested by trypsinization and stained with 0.2% trypan blue and then were counted using a hemacytometer under the light microscopy. Trypan blue penetrated cells were considered dead, whereas trypan blue excluded cells were considered to be viable. Each assay was carried out for triplicate and the concentration of the SVT which causes 50% reduction in viable cell count (IC_50_) were calculated by dividing number of viable cells in treated group to the number of viable cells in control (untreated) group (17).

*Lactate dehydrogenase (LDH) release assay*

Cytolysis was assessed by determining the release of the cytosolic enzyme lactate dehydrogenase. Briefly, Cells were seeded in 96-well plates at the density of 1×10^4^ cells/well in culture medium. following overnight incubation, the medium was replaced with serum free medium and cells were exposed to various concentrations of venom. Some wells were exposed to the different concentrations of cisplatin as a positive control. Cells were incubated for 24 h and LDH activity was measured in the supernatants using the *in-vitro* toxicology assay kit (Cytotoxicity Detection Kit, Cat. No.1644793, Roche, United States) according to manufacturer’s instructions. Spectrophotometric absorbance of the colored formazan was determined using the microplate reader at 490 nm wavelength and 690 nm reference wave length. Reference controls for 0% (low control) and 100% (high control) cytolysis consisted of medium of untreated cells and medium from cells incubated with 0.1% (v/v) of Triton X-100, respectively. All assays were repeated in triplicate.

*Fluorescence microscopic analysis of cell death*

To further investigate on the mode of cell death caused by this venom, apoptotic and necrotic cells were determined using acridine orange/ethidium bromide double staining method as described by Niknafs and Shirazi in 1998. Acridine orange is taken up by both viable and nonviable cells and emits green fluorescence. Ethidium bromide is taken up only by nonviable necrotic cells and emits red fluorescence due to the dye intercalation into DNA. Briefly, after the exposure time, cells were harvested by trypsinization, centrifuged at 700g, washed with PBS and treated with ethidium bromide (EtBr) and acridine orange (AO) solution (both 25µg/mL in PBS) and finally observed under a Moticam Pro 2828 fluorescence microscope (18).

Treated cells were quantitated according to the following descriptions: (A) normal nuclei (bright green chromatin with organized structure), (B) early apoptotic (bright green chromatin that is highly condensed or fragmented), (C) late apoptotic (bright orange chromatin that is highly condensed or fragmented) and (D) necrotic (Deep saturated red cells). At least 200 cells from randomly selected fields were counted and quantitated for each data point. The apoptotic index (percentage of apoptotic cells) was calculated as the number of apoptotic cells/total cells counted multiplied by 100 (19).

*Flow cytometric analysis of apoptosis*

Propidium iodide (PI) stained cells flow cytometry was carried out to quantify the apoptotic cells (20). Briefly, Cells were seeded in 25 T flask, and treated with various concentrations of SVT for 24 and 48 h. Cells were then harvested by trypsinization, collected by centrifugation (700 *g* for 10 min at 4 °C), washed twice with ice-cold PBS and collected again by centrifugation. Cells were then fixed in 70% (v/v) ethanol at 4 °C for 30 min. After fixation, cells were centrifuged and resuspended in 1 mL buffer (100 µg /mL RNase A, 500 µg/mL propidium iodide in PBS) at 37 °C for 30 min. Cells were detected using a flow cytometry (Partec-CyFlow space) using 620 nm filter for PI detection, and analyzed by software program (Partec-FloMax, USA). The cell cycle distribution and proportion of the sub-G1 group (apoptosis) were determined and analyzed. 

*Flow cytometric detection of mitochondrial membrane potential*

Changes in mitochondrial membrane potential were studied with the use of Rhodamine123 (Rh123). Approximately 5x10^5^ HepG2 cells/mL in 12-well plates were treated with 2.5 µg/mL of SVT for 24 h. At the end of treatment, cells were washed twice with PBS, incubated with Rhodamine 123 (0.5 µM) in the culture medium for 30 min at 37°C and then analyzed using a flow cytometry as described previously (21).

*Caspase-3 activity assay*

Caspase-3 activity in the treated and control cells, was measured using a caspase-3 colorimetric assay kit (Sigma, St Louis, MO, USA) according to the manufacturer's instructions. The kits utilize synthetic tetrapeptides labeled with p-nitroanilide. Briefly, 1×10^6^ cells were treated with different concentration of SVT (0, 2.5, 5, 7.5, 10 and 15 µg/mL) for 24 h. In addition, 1×10^6^ cells were treated with 5 µg/mL SVT for 12, 24, 48 and 72 h. Then the cells were lysed in the supplied lysis buffer. The supernatant was collected and incubated with the supplied reaction buffer containing dithiothreitol and substrates at 37°C. The reaction was measured by changes in absorbance at 405 nm using a microplate reader (BioTek-EL808, United States). The result of control groups were set as 100% activity and test groups were compared to this value.

*Statistical analysis*

The data were expressed as mean±standard deviation (SD) of three independent experiments. Wherever appropriate, the data were subjected to statistical analysis by non-parametric linear regression, Student’s t-test and one-way analysis of variance (ANOVA) followed by Dunnett’s method for multiple comparisons test. A value of *p*<0.05 was considered significant. All statistical analyses were performed using GraphPad Prism^®^ version 5.00 for Windows (GraphPad^®^ Software, San Diego, CA).

## Results

*Morphological changes*

**Figure 1:**
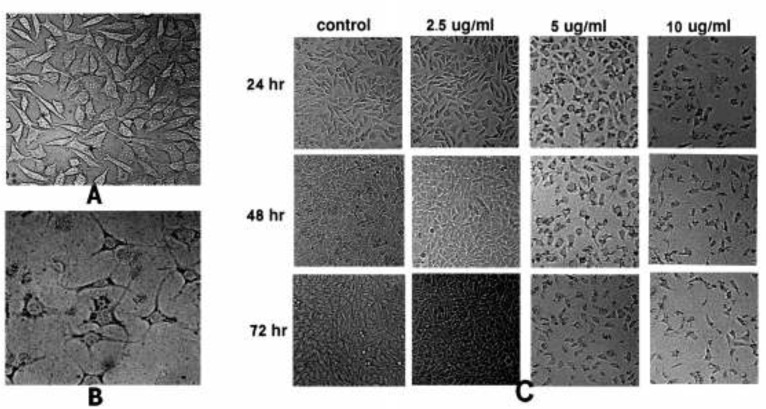
Morphological alterations of HepG2 cells after exposure to different concentrations of SVT, observed by normal inverted light microscopy. (A) Untreated cells. (B) Morphological changes in HepG2 cells after 24 h treatment with 10 µg/mL of SVT. Detachment of cells from the dish, cell rounding, cytoplasmic blebbing, chromatin condensation and irregularity in shape are observable. (C) Significant decrease in attached cell population after treatment with different concentrations of SVT for various time periods

To observe the effect of SVT on cell morphology, treated HepG2 cells were examined by normal inverted light microscopy and compared with untreated cells. Treated cells showed significant changes in comparison to the untreated cells. As shown in Figure 1B, treated cells were detached from the dish surface and cell rounding, cytoplasmic blebbing, chromatin condensation and irregularity in shape was observable. Significant decrease in cell count was seen by the increase in SVT concentration and time of treatment (at the concentrations above 5 µg/mL). On the contrary, untreated cells remained confluent throughout the incubation period (Figure 1C).

*Cell Proliferation Assays*

**Figure 2:**
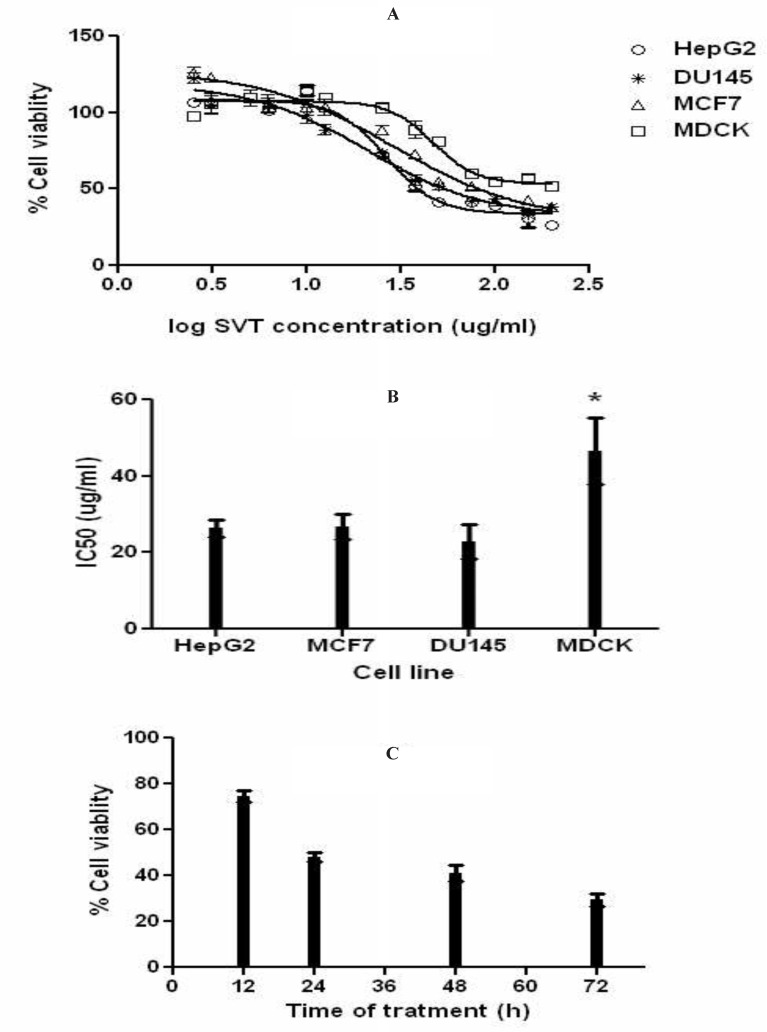
Effects of SVT on cell proliferation (MTT assay). The cells were treated with different concentrations of SVT for 24 h. At the end of the incubation time, cell viability was determined by the MTT reduction assay as described in methods section. (A) Dose-response curve for inhibition of cell proliferation in different cell lines. (B) IC_50_ of SVT in different cell lines. (C) HepG2 cells were treated with IC_50_ concentration for 72 h and percentage of viable cells was determined at 12, 24, 48 and 72 h. The data are expressed as mean±SD of three independent experiments

The result of MTT assay showed that SVT inhibits cell proliferation in a dose and time-dependent manner. Figure 2A shows the dose–response cell viability curves for HepG2, MCF7, DU145 and MDCK cells. As shown in this Figure, SVT inhibits cell proliferation in all cell types by the same manner. As shown in Figure 2B, IC_50_ values of SVT in these three cancer cell lines were close to each other (26.59, 28.85 and 21.17µg/mL for HepG2, MCF7and DU145 cell lines, respectably) and showed no significant difference, but significantly (*P*<0.01) differ from the MDCK normal cell line (IC_50_=47.1 µg/ml). The trend of increasing cytotoxicity by increasing incubation time was observed in HepG2 cell line and results are shown in Figure 2C. 

*Fluorescence microscopic analysis of cell death*

**Figure 3:**
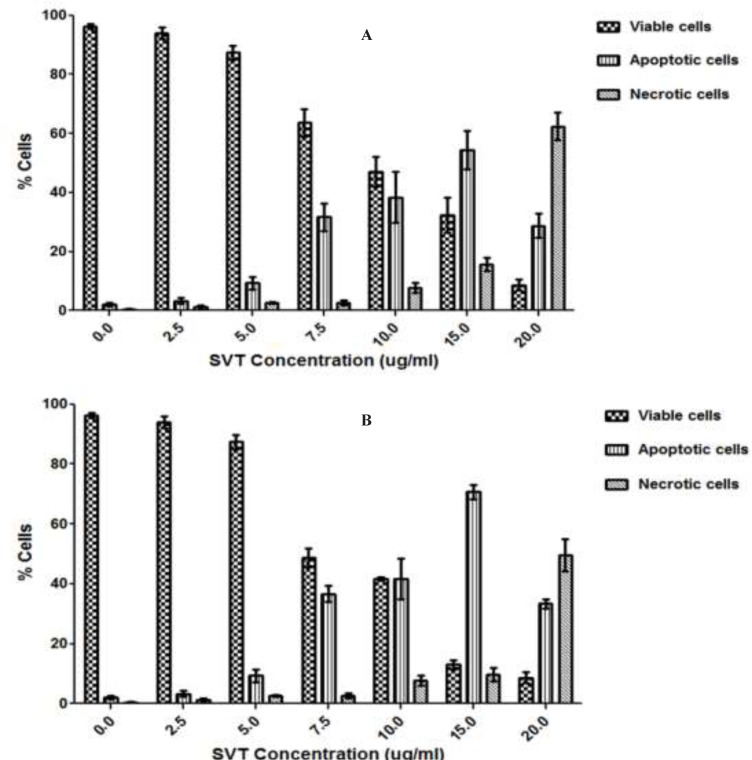
Fluorescence microscopic analysis of cell death. HepG2 cells were treated with different concentrations of SVT for 24 and 48 h. Then the cells were harvested and stained with Acridine orange and ethidium bromide and were observed under fluorescence microscopy. 200 cells randomly were counted and percentages of viable, apoptotic and necrotic cells were calculated. Apoptotic cell death increased significantly (*P*<0.05) in a time and dose-dependent manner. However, no significant (*P*<0.05) difference was observed in the count of necrotic cells at concentrations below 10 µg/mL of SVT. (A) 24 h treatment. (B) 48 h treatment

Using fluorescence microscopy, 200 cells were counted arbitrarily and compared to the untreated negative control. The study revealed that SVT induced morphological features that relate to apoptosis in a time-dependent manner. Early apoptotic cells were obvious by intercalated AO-DNA within the cell nuclei. These cells had green nuclei, but nuclear chromatin condensation was visible as bright green patches or fragments. In contrast, untreated cells were observed with a green intact nuclear structure. 24 h after treatment, blebbing and nuclear chromatin condensation were noticed (as an indication of moderate apoptosis). In addition, in the late stages of apoptosis, changes such as the presence of reddish-orange color due to the binding of EtBr to denatured DNA were observed. Differential scoring of treated cells (200 cells populations) after 24 and 48 h of treatment showed statistically significant differences (*P*<0.05) in apoptotic cells compared to the controls at doses above 5 µg/ml, which indicated clearly that a time-dependent apoptotic effect had occurred. There was no statistically significant difference (*P*<0.05) in necrotic populations at concentrations below 10 µg/ml, however at doses above 15 µg/mL necrotic cell population was significantly increased. The best apoptotic/necrotic cells ratio was seen at 15 µg/mL after 48 h of treatment (Figure 3).

*Trypan Blue Exclusion Assay*

**Figure 4:**
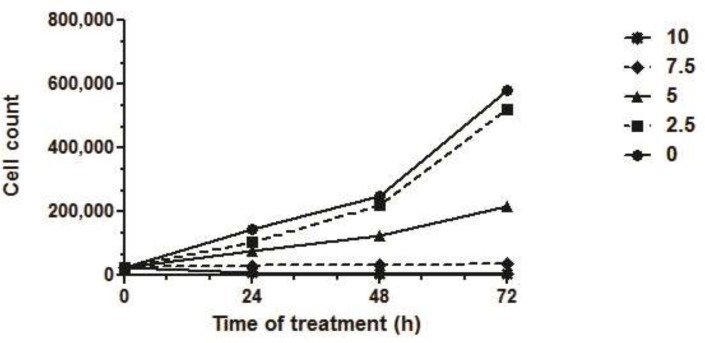
Trypan blue cytotoxicity assay. The cells were treated with different concentrations of SVT for 24, 48 and 72 h, harvested by trypsinization and stained with 0.2% trypan blue. Viable cell were counted under light microscope. Values are means ± S.D of three independent experiments

Trypan blue exclusion assay showed that SVT increased cell death in a dose and time-dependent manner (Figure 4). Analysis of cell survival showed that the IC_50_ of SVT on HepG2 cells was 5.04, 4.94 and 4.45 µg/mL after 24, 48 and 72 h of treatment, respectively. There was no significant difference between IC_50_ values of 24 and 48 h treatments, while IC_50_ of 72 h. treatment was statistically different from the calculated IC_50_ after 24, 48 h (Figure 4).

*Lactate Dehydrogenase Release (LDH) Assay*

**Figure 5:**
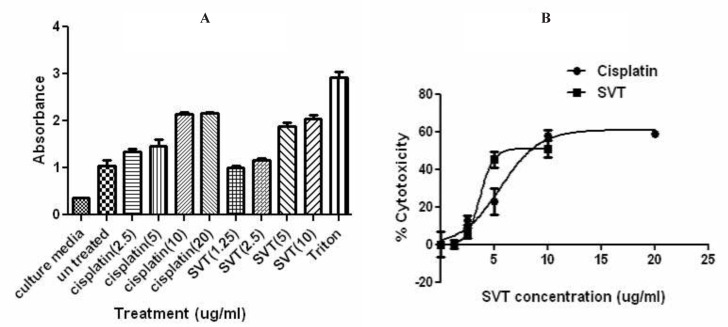
Effect of SVT and Cisplatin on LDH leakage in HepG2 cells. The cells were treated with different concentrations of SVT and Cisplatin for 24 h. At the end of the incubation period, the LDH assay was performed to assess the LDH leakage as described in methods section. No significant difference was observed in LDH leakage at 10 µg/mL of SVT and cisplatine (*P*<0.05). The data are expressed as mean±SD of three independent experiments

For the study of the lytic behavior of SVT on tumor cells and its comparison with a standard chemotherapeutic agent, HepG2 cells were treated with different concentrations of SVT and cisplatin, simultaneously. Results are presented in Figure 5. Dose-dependent increase in LDH release was seen in both treatment groups. This increase was significantly higher than negative control group (untreated cells) and lower from positive control group (Triton-exposed cells). LDH release in untreated cells and cells that were exposed to 1.25 µg/mL SVT showed no significant differences. The absorbance value of the cells that were treated with 10 µg/mL SVT and 10 µg/mL cisplatin showed no significant differences. This pattern also applies to 2.5 µg/mL but not to 5 µg/mL of these agents. 

*Flow cytometric analysis of apoptosis*

**Figure 6:**
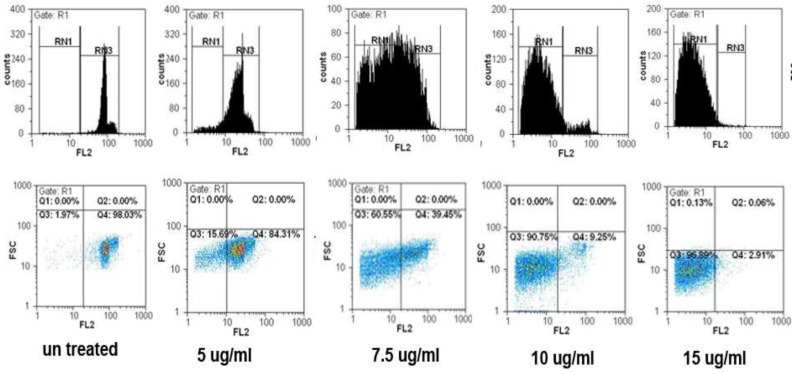
SVT induced cell cycle arrest and apoptosis in HepG2 cells. The cells were treated with 0,5, 7.5, 10 and 15 µg/mL of SVT for 48 h. They were then harvested and stained with PI. Cell cycle, sub-G1 groups and apoptotic populations were examined by flow cytometry as described in methods section. Upper panels represent profiles of cell cycle, distribution of cells in the cell cycle and proportion of the sub-G1 cells. Lower panels show the proportion of apoptotic and normal cells

The results from flow cytometric analysis of cell death in the present study showed a mark increase in apoptotic cell populations in HepG2 and MCF7 cells after treatment with SVT in time and dose-dependent manners. As described previously cells were treated with different concentrations of SVT for 24 and 48 h and cell death was analyzed by flow cytometry. As shown in Figure 6 percentages of apoptotic cells were elevated by the increase in SVT concentration (1.55, 21.43, 60.98, 90.48 and 97.38% for 0, 5, 7.5, 10 and 15 µg/mL of SVT, respectively). A large and extensive arrest in all phases of the cell cycle was obvious after exposure to 7.5 µg/mL of SVT. Apoptotic cell populations were increased by the change of exposure time from 24 to 48 h. The results showed that SVT induced G0/G1 arrest and promoted the S-phase fraction and increased the proportion of cells in the sub-G1 group (apoptosis). These results demonstrated that SVT has potent apoptotic effects in tumor cells.

*The effect of SVT on the mitochondrial membrane potential (MMP)*

**Figure 7:**
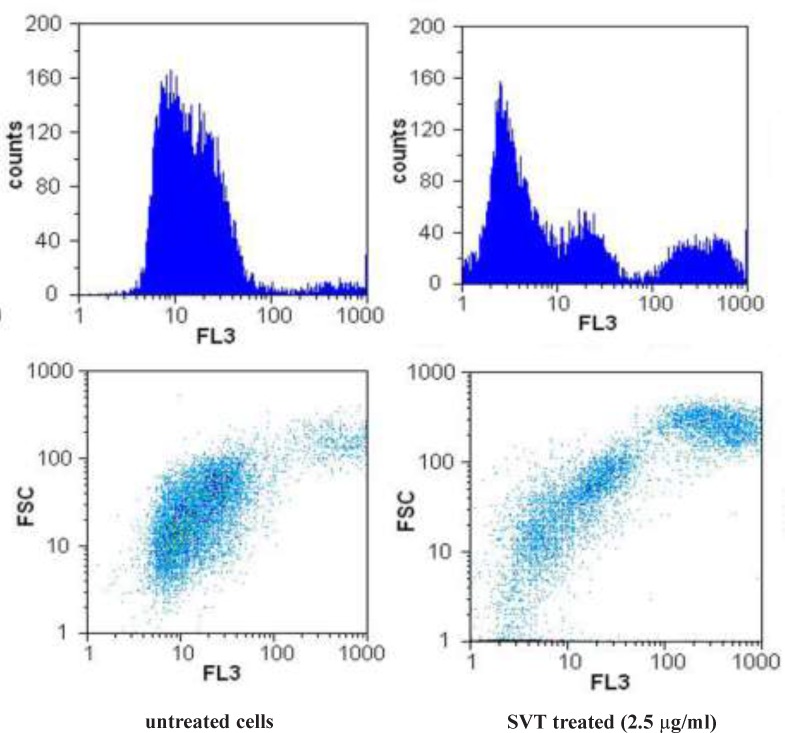
Effect of SVT on mitochondrial membrane potential (MMP) in tumor cells. The cells were incubated with SVT (2.5 µg/mL) for 24 h. At the end of incubation, the medium was removed, and cells were washed twice with PBS, the cells were incubated with Rhodamine123 (0.5 µM) for 30 min at 37 ˚C in the dark. The cells were harvested and suspended in PBS. The MMP was measured by the fluorescence intensity of 20000 cells by flow cytometer

To study the possibility that SVT might induce apoptotic cell death in tumor cells through disruption of MMP, we examined changes of MMP after incubating cells with SVT by measuring differences in fluorescence between control and SVT-treated cells. Upon mitochondrial permeability transition pore opening, mitochondria lost their membrane potential across the inner membrane. Snake venom toxins consistently and rapidly depolarized mitochondria, as reflected by a decrease in the fluorescence intensity (Figure 7). As seen in this Figure, low concentrations of SVT that could not induce remarkable cytotoxic effects, can decrease the MMP significantly. This result suggests that snake venom toxins causes dissipation of MMP and disruptions in mitochondrial functions, which might be involved in the mechanisms of snake venom toxins-induced apoptotic cell death in tumor cells. 

*Caspase-3 activity assay*

**Figure 8:**
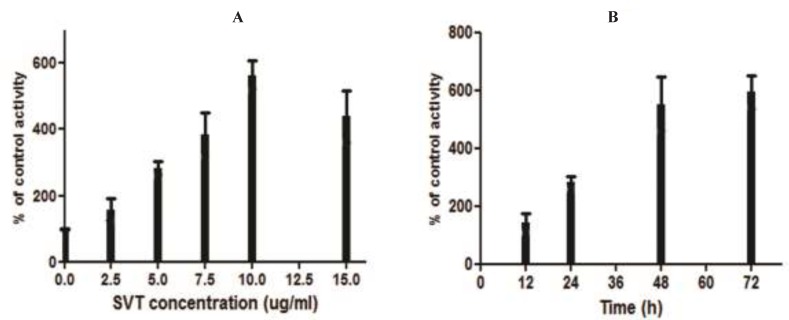
Measurement of caspase-3 activity. (A) The cells were treated with different concentrations of SVT for 24 h and caspase-3 activity levels were measured by a commercial kit (Sigma). Mark increase in caspase-3 activity was seen by increase in SVT concentrations. (B) The cells were treated with 5 µg/mL of SVT for 72 h and caspase-3 activity levels were measured in 12, 24. 48 and 72 h. A time-dependent pattern was observed in caspase-3 activity

The colorimetric assay indicated that SVT promoted the caspase-3 activation in a time and dose-dependent manner (Figure 8). As shown in this Fgure, caspase-3 was significantly activated at doses higher than 2.5 μg/mL of SVT and its maximal activity was shown at 10 μg/ml.

## Discussion and Conclusions

Cancer is the major public health burden in all countries. Currently, 1 of 4 deaths in U.S. is due to cancer. Since cancer is the leading cause of death worldwide, there is an urgent need for finding a better way to treat it. Various therapies have been used for treating cancer such as chemotherapy, radiotherapy, immunotherapy and gene therapy (22). Between these various therapeutic methods, chemotherapy remains the predominant option but, unfortunately, patients eventually become resistant to the therapy. Meanwhile, search for newer anticancer agents especially from natural products is increasing, and animal venom has been shown to have a wide spectrum of biological activities. Multicomponency and biodiversity of venoms make them unique tools for new therapeutic agent development. Between various type of venomous animals, snakes, because of their widespread distribution, considerable volumes of venom and several bioactive components have attracted much attention. Early reports about anticancer effects of snake venom toxins date back to 1933 (4). After that valuable work, several studies on the SVT began. The anticarcinogenic activities of crude venom of Indian monocellate Cobra (*Naja kaouthia*) and Russell’s viper (*Vipera russelli*) were studied on carcinoma, sarcoma and leukemia models (23). Some other studies showed that the snake venom toxins from *Vipera lebentina turnica* could induce apoptotic cell death in human prostate cancer cells, neuroblastoma cells and colon cancer cells. This toxins could increase the expression of pro-apoptotic protein Bax and Caspase-3, but down-regulates the anti-apoptotic protein Bcl-2 (8, 24-25). In case of *in-vivo* study also snake venom showed potent cytotoxic and anticancer effects on different types of cancer (26-27). Studies of Strizhkov and colleagues (1994) showed that neurotoxin II form venom of *Naja naja oxiana* could induce apoptosis in Mouse Fibroblast cell line (L929) and Human Erythroleukemic cell line (KS62) (12). These results were obtained by only a DNA fragmentation assay and not any other features of mode of cell death.

Results presented in this study showed that crude venom of *Naja naja oxiana* can induce cytotoxicity and apoptosis in different cancer cell lines in a dose-dependent manner. In microscopic observations apoptotic patterns of cell death such as cell rounding, cytoplasmic blebbing, and chromatin condensation were clearly observed. Induction of apoptosis is the most important mechanism for many anticancer agents. In fact, an ideal anticancer agent mostly potentiates apoptotic effects in cancer cells, with minimum necrotic effects (28). Fluorescence microscopic analysis of cell death in this study showed that treatment of HepG2 cells with SVT at concentrations below 15 µg/mL induce more apoptotic cell death rather than necrotic death. A very good arrest of cells in all phases of the cell cycle and the best ratio between apoptotic and necrotic death was seen in 15 µg/mL of SVT. The portion of necrotic death increases rapidly with SVT concentrations above 20 µg/mL compared to apoptotic cells. A comparison of these behaviors with a standard anticancer agent is done using cisplatin on HepG2 cells. The result (data not shown) presents a stronger apoptotic induction characteristic for SVT compared to the cisplatin on this cancer cell line. 

MTT cell proliferation assay indicates that treatment of different cancer cell line with various concentrations of SVT inhibits cell proliferation. This inhibition significantly differs from the normal cell line (calculated IC_50_ for normal cell line was about two-times higher than cancer cell lines). As seen in Figure 2 low concentrations of SVT results in significantly increased cell proliferation. To explain this phenomenon, it is necessary to remember that MTT assay, assess cell viability by evaluation of dehydrogenase enzyme activity and reduction of tetrazolium dye. SVT contains different classes of active enzymes that could considerably reduce tetrazolium dye and affect the results of MTT assay. Along with increasing SVT concentration, cell viability significantly decreases and inhibition of cell proliferation increases. Calculated IC_50_ for SVT by another method (about 5.1 and 3.9 µg/mL IC_50_ values calculated by trypan blue exclusion assay and lactate dehydrogenase release assay, respectively) confirms that SVT induces cell death at very low concentrations comparable to some anticancer drugs. Furthermore, SVT shows no significant membrane lytic effects compared to cisplatin at apoptotic concentrations (Figure 5).

Mechanism of apoptosis induction is another important question that had to be responded in this study. Apoptosis is the cascade of molecular events leading to cell death. In general, apoptosis is mediated through two major pathways, the extrinsic (death receptor-mediated) and intrinsic (mitochondrial-mediated) pathways (29). In the intrinsic apoptotic cell death pathway, oxidative damage of cells is the critical regulator of apoptotic cell death. Reactive oxygen species induce opening of the permeability transition pore of mitochondria (MMP) by oxidation-dependent mechanisms (30). Loss of membrane potential can cause release of cytochrome*c* from mitochondria, decrease in the Bcl-2/Bax ratio and finally apoptotic cell death. Measurement of changes in mitochondrial membrane potential in SVT treated cells indicate that SVT can induce significant loss of MMP in various cancer cell lines even at low concentrations (about 2.5 µg/ml). These finding suggest that SVT induces apoptosis in cancer cells via intrinsic pathway and mitochondrial damage.

Although there are many characters for apoptosis, however the key hallmark for apoptosis is the activation of caspase and dysfunction of mitochondria (Ip *et al*. 2008). Caspases are a family of cysteine proteases that have essential roles in apoptotic pathways; caspase-3 in particular, has many cellular targets and, when activated, produces the morphologic features of apoptosis. Many studies have determined that a variety of chemotherapeutic agents induce apoptosis through the activation of caspase-3 (Moon *et al* 2006). Our data indicate that caspase-3 plays an important role in SVT-induced apoptosis in cancer cells and that apoptotic cell death is accompanied by significant caspase-3 activation.

The study by Feofanov collogues on Caspian cobra venom cytotoxins revealed that Cytotoxins I and II easily penetrate into living cancer cells and accumulate markedly in the lysosomes, suggesting the lysosomal damage to be the cause of cell death induced by these toxins (13). With regard to recent advance in recognition of apoptotic pathways and the role of lysosomal damages and release of cathepsins in the activation of proapoptotic proteins such as Bcl-2, Bid and Bax (31), our results confirm Feofanov findings and apoptogenic potential of SVT.

As a conclusion of what is presented in this study, *Naja naja oxiana *venom is a potent inducer of apoptosis in cancer cell lines with minimum effects on normal cell. This potential might candidate this venom as a suitable choice for cancer treatment, which needs to be further explored in complementary studies.
